# Reprogramming of leukemic cell metabolism through the naphthoquinonic compound Quambalarine B

**DOI:** 10.18632/oncotarget.21663

**Published:** 2017-10-07

**Authors:** Karel Vališ, Valéria Grobárová, Lucie Hernychová, Martina Bugáňová, Daniel Kavan, Martin Kalous, Jiří Černý, Eva Stodůlková, Marek Kuzma, Miroslav Flieger, Jan Černý, Petr Novák

**Affiliations:** ^1^ BIOCEV, Institute of Microbiology, v.v.i., The Czech Academy of Sciences, Vestec, Czech Republic; ^2^ Department of Biochemistry, Faculty of Science, Charles University, Prague, Czech Republic; ^3^ Department of Cell Biology, Faculty of Science, Charles University, Prague, Czech Republic; ^4^ Institute of Microbiology, v.v.i., The Czech Academy of Sciences, Prague, Czech Republic; ^5^ Faculty of Chemical Technology, University of Chemistry and Technology, Prague, Czech Republic; ^6^ BIOCEV, Institute of Biotechnology, v.v.i., The Czech Academy of Sciences, Vestec, Czech Republic

**Keywords:** metabolism, leukemia, naphthoquinones, mitochondria, therapy

## Abstract

Abnormalities in cancer metabolism represent potential targets for cancer therapy. We have recently identified a natural compound Quambalarine B (QB), which inhibits proliferation of several leukemic cell lines followed by cell death. We have predicted ubiquinone binding sites of mitochondrial respiratory complexes as potential molecular targets of QB in leukemia cells. Hence, we tracked the effect of QB on leukemia metabolism by applying several omics and biochemical techniques. We have confirmed the inhibition of respiratory complexes by QB and found an increase in the intracellular AMP levels together with respiratory substrates. Inhibition of mitochondrial respiration by QB triggered reprogramming of leukemic cell metabolism involving disproportions in glycolytic flux, inhibition of proteins O-glycosylation, stimulation of glycine synthesis pathway, and pyruvate kinase activity, followed by an increase in pyruvate and a decrease in lactate levels. Inhibition of mitochondrial complex I by QB suppressed folate metabolism as determined by a decrease in formate production. We have also observed an increase in cellular levels of several amino acids except for aspartate, indicating the dependence of Jurkat (T-ALL) cells on aspartate synthesis. These results indicate blockade of mitochondrial complex I and II activity by QB and reduction in aspartate and folate metabolism as therapeutic targets in T-ALL cells. Anti-cancer activity of QB was also confirmed during *in vivo* studies, suggesting the therapeutic potential of this natural compound.

## INTRODUCTION

Cancer cells represent rapidly dividing population of cells with a number of different metabolic requirements, which exemplify potential targets for cancer therapy. Alterations in cancer cell metabolism were observed for the first time by German biochemist Otto Warburg in 1920s. He described that cancer cells rely on glycolytic metabolism even in the presence of oxygen and produce lactate as glycolytic end product [1]. Based on these observations, Warburg hypothesized that cancer cells carry dysfunctional mitochondria [2]. On the other hand, under physiological conditions, some proliferating cell types were found to preferentially rely on glucose metabolism without any mitochondrial defect [3].

Pyruvate, an end product of glycolytic pathway, mainly supplies mitochondrial metabolism. Importantly, metabolic pathways derived from glycolysis also provide intermediates for the synthesis of amino sugars, amino acids, and nucleotides. Recent reports point out decreased activity of pyruvate kinases (PKM) in cancer cells due to preferential expression of PKM2 isoform driven by C-MYC oncoprotein and inhibition of pyruvate dehydrogenase (PDH) activity, as a result of inhibitory phosphorylation catalyzed by PDH kinase (PDK) [4, 5]. These events may lead to inhibition of mitochondrial complex I activity in the cancer cells. PKM2 exists in cancer cells as a dimer with lower catalytic activity, which results in the accumulation of glycolytic intermediates and their subsequent transformation in biosynthetic pathways [6]. Hence, increased glycolysis contributes to the biosynthesis of building blocks indispensable for the proliferation of cancer cells. Biosynthetic pathways utilizing glycolytic intermediates involve hexosamine synthetic pathway, pentose pathway, and serine/glycine synthetic pathway, which regulates folate metabolism and is involved in purine synthesis and mitochondrial metabolism [7]. Consistently with these findings, activators of PKM2 activity act as tumor suppressors [8]. Recent studies suggest an important role of mitochondria in cancer cell proliferation due to the synthesis of aspartate from oxaloacetate and dependency of cancer cells on glutamine supplementation [9–11]. In this logic, cancer cells utilize conversion of glutamine to alpha-ketoglutarate (AKG) as a major source of metabolites, which feed oxaloacetate synthesis and act as a source of electrons for electron transport chain (ETC) orchestrated by the complex II activity. This switch in cancer cell metabolism is also linked to C-MYC activity. Several signaling pathways were recently identified as regulators of C-MYC expression and cancer cell metabolism. These pathways involve AMPK and Hippo signaling pathways, which can act as potent tumor suppressors [12, 13].

Biosynthetic pathways represent a promising target for cancer therapy. Purine synthesis pathway is one of the first metabolic pathways targeted for cancer therapy. Purine synthesis can be inhibited using compounds interfering with folate metabolism (anti-folate), which represents an important cofactor in purine synthesis [14]. Another example of pioneer cancer therapy targeting metabolism is depletion of asparagine (and glutamine) using asparaginase. This therapy utilizes dependency of several cancer cell types on asparagine and glutamine and thus, inhibits cancer cells proliferation followed by cancer cell death. On the other hand, asparaginase treatment triggers reprogramming of cancer cell metabolism toward fatty acid oxidation, providing the support of tricarboxylic acid (TCA) cycle intermediates. Consistent with these findings, the inhibitors of fatty acid oxidation leads to an increase in the sensitivity of cancer cells to asparaginase treatment [15]. Other metabolic vulnerabilities of cancer cells are reviewed by Vander Heiden [16].

Quinones represent natural aromatic compounds participating in key metabolic processes nearly in all living organisms and can be exemplified by ubiquinone (UbQ). This molecule acts as a key electron transporter during several biochemical reactions involved in cellular energetic and biosynthetic pathways. UbQ is structurally related to naphthoquinones, which are synthesized in a large spectrum of organisms involving bacteria, plants, and fungi. Moreover, 1,4-naphthoquinones (NQ) represent an important group of anti-cancer drugs commonly used in clinical practice, mainly acting as redox cyclers and alkylating agents [17]. The oxidoreduction of quinone induces reactive oxygen species (ROS) production followed by cancer cell death [18]. NQ can be reduced by several NADH or NADPH oxidoreductases and can also interfere with their specific functions. Several NQs were described as potent inhibitors of mitochondrial respiratory complexes disturbing the energetic and metabolic balance of the cell [19–21]. NQ carrying non-substituted C2 or C3 position may react with thiols or amines of biomolecules to form covalent adducts. These NQ-based covalent modifications often interfere with proper functioning of target molecules as in the case of protein tyrosine phosphatase inhibition triggered by covalent modification of active cysteine residue [22].

We have recently identified a group of novel and natural naphthoquinonic compounds with potential anti-cancer activity and confirmed anti-leukemic activity of the novel compound 1,4-naphthoquinone Quambalarine B (QB). QB effectively blocks proliferation of several leukemic cell lines and induces loss of proton gradient across the inner mitochondrial membrane [23]. QB also inhibits expression of C-MYC oncoprotein and attenuates mTOR signaling in several tested leukemic cell lines [24]. Since QB possesses substituted C2 and C3 positions in its structure, we presume redox cycling together with target oxidoreductases interference as a key mechanism responsible for QB anti-cancer activity. Hence, we employed several proteomic and metabolomic techniques to uncover the molecular mechanism behind the QB effect on the metabolism of leukemic cells. We suggest a model of metabolic reprogramming, which involves inhibition of mitochondrial respiration, accumulation or inhibition of specific metabolites and amino acids, and imbalance in the regulation of glycolysis and glycolysis-derived biosynthetic pathways. QB-driven rearrangement of leukemic metabolism leads to inhibition of proliferation partially through the simulation of nutrient starvation. Importantly, we confirmed QB anti-tumor activity *in vivo* using mouse xenograft model verifying QB as a new promising anti-cancer drug. Finally, our model provides novel and complex insight into the metabolism regulatory network in leukemic cells and highlights the novel metabolic circuits representing new promising targets for leukemia treatment.

## RESULTS

### QB acts as a competitive inhibitor of ubiquinone binding on complex I and complex II

**Figure 1:**
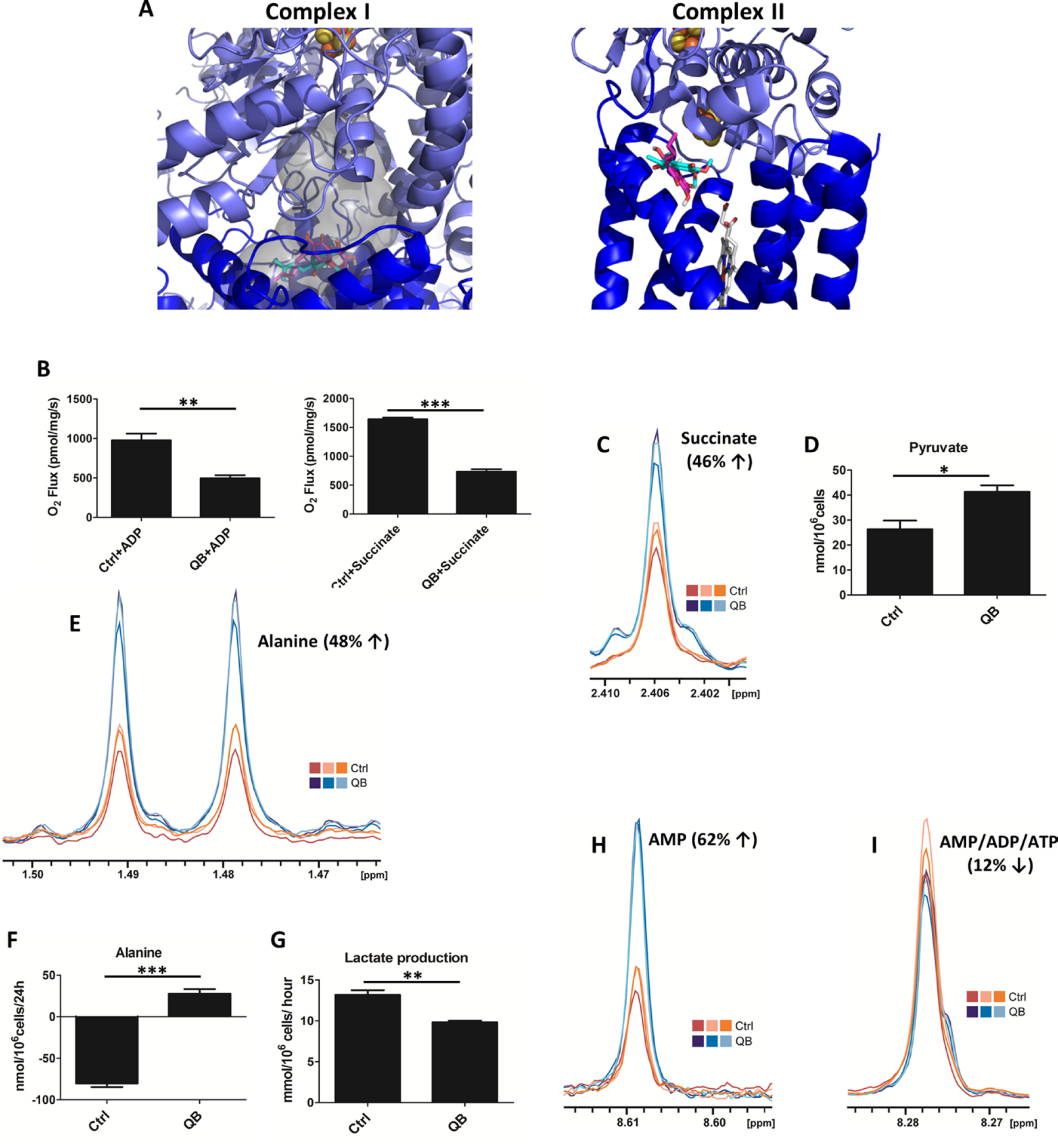
Quambalarine B (QB) inhibits the activity of mitochondrial complexes I and II in Jurkat cells (**A**) Molecular docking of QB to ubiquinone binding site in mitochondrial complex I and complex II. The grey surface corresponds to a potential ubiquinone binding cavity as obtained by analysis of the crystal structure by 3V program. (**B**) Effect of QB (20 µmol/L) on the activity of individual mitochondrial complexes determined by oxygen consumption. Oxygen uptake in isolated rat mitochondria is expressed as pmol/s/mg protein. (**C**) Levels of succinate in control (orange lines) and QB-treated (blue lines) cells determined using NMR analysis. (**D**) Levels of pyruvate in control (ctrl) and QB-treated cells (QB) determined by enzymatic assay. (**E**) Levels of intracellular alanine in control (orange lines) and QB-treated cells (blue lines) determined by NMR analysis. (**F**) Changes in alanine levels in the culture medium of control (ctrl) and QB-treated cells (QB) after 24 h of incubation determined by HPLC analysis. (**G**) Lactate production by control (ctrl) and QB-treated cells (QB) determined by enzymatic assay. (**H**) AMP levels in control (orange lines) and QB-treated (blue lines) cells determined by NMR analysis. (**I**) AMP/ADP/ATP levels in control (orange lines) and QB-treated (blue lines) cells determined by NMR analysis. Data are shown as means from three independent experiments ± SEM. ^*^, significant differences with *P* ≤ 0.05. ^**^, significant differences with *P* ≤ 0.01. ^***^, significant differences with *P* ≤ 0.001.

Reflecting structural similarity, we focused on a key electron transporter UbQ as a possible target to reveal the molecular mechanism behind the QB effects on mitochondrial function. We predicted binding mode for UbQ and QB docking to mitochondrial respiratory CI and CII structures using molecular modeling strategy. For the CII structure, where the UbQ position was known from crystal structures, we could compare the performance of used docking algorithm. The crystal orientation was reproduced by the second pose (with the predicted binding affinity for the slightly tilted first pose being only 0.1 kcal/mol more favorable). The predicted QB binding site overlapped with the UbQ position and its binding was more favorable by about 0.5 kcal/mol. In the case of CI, both ligands shared a binding site within the expected UbQ binding cavity, and again QB binding affinity was more favorable compared to UbQ by about 0.5 kcal/mol. This suggests that QB may affect UbQ interactions with respect to CI and CII structures (Figure 1A), being the higher affinity interactor.

### QB treatment inhibits activity of mitochondrial complexes I and II

To validate the possible competitive inhibitory effect of QB on the activity of mitochondrial respiratory complexes *in vivo*, the mitochondria isolated from rat hepatocytes were used as a model system, and oxygraphy was used as an experimental approach. We observed significant inhibition of complexes I and II activity during QB incubation with isolated mitochondria compared to mitochondria incubated with DMSO as a control, suggesting a possible UbQ competitive mechanism underlying QB bioactivity (Figure 1B). The respiratory ratio was significantly lower in mitochondria incubated with 20 µmol/L QB (1.3) compared to control (4.28). To continue to the whole cell system, we estimated the inhibitory concentration (IC_50_) of QB toward oxidoreductases in leukemia cells by MTS assay. Using this method, we calculated IC_50_ = 2 µmol/L, and this concentration of QB was used in all following experiments (Supplementary Figure 1). Inhibition of individual respiratory complexes often resulted in the accumulation of input metabolites, i.e., pyruvate and succinate [10, 25]. We confirmed increased levels of both metabolites in leukemia cells after QB treatment using NMR and enzymatic techniques, respectively (Figure 1C and 1D). Moreover, at least two other metabolic pathways are known to drive pyruvate transformation in cancer cells. Pyruvate can be reduced to lactate by the cytosolic lactate dehydrogenase (LDH) activity or transaminated into alanine by mitochondrial alanine transaminase activity, which also produces AKG from glutamate. We tracked pyruvate metabolic pathway after QB treatment on the basis of alanine level estimation using NMR, enzymatic and HPLC techniques. We confirmed significant changes in alanine and lactate concentrations. We detected significant increase for intracellular alanine as well as for alanine in the culture medium, suggesting its transport out of the cells (Figure 1E and 1F). On the other hand, the intracellular concentration of lactate remained constant (data not shown), but it decreased extracellularly, suggesting inhibition of lactate synthesis and transport after QB treatment (Figure 1G). Finally, we determined cellular ATP and AMP concentrations after QB treatment using NMR analysis. We observed a slight decrease in signal common to all adenosine nucleotides, but on the other hand, the strong intensity was found in the signal belonging solely to AMP. These results clearly showed an increase in AMP fraction from all adenosine nucleotide variants (AMP+ADP+ATP; Figure 1H and 1I). These observations can play an important role in global QB-driven reprogramming of cancer metabolism since lactate-driven acidification of cytosol and alanine levels represent well-known regulators of glycolytic flux.

### QB treatment has insignificant effect on transport of glucose into leukemia cells

**Figure 2:**
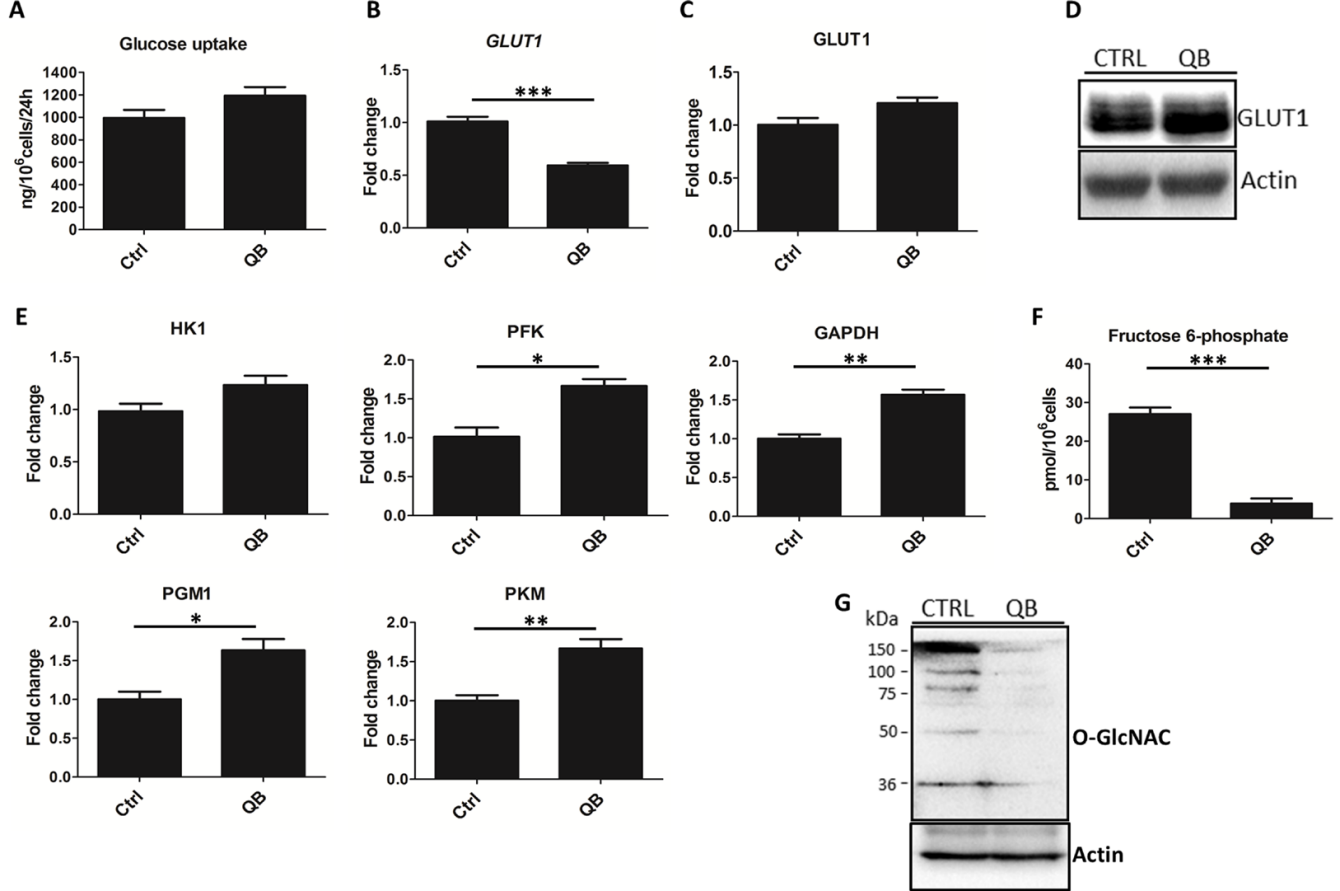
Quambalarine B (QB) treatment has a strong impact on glycolysis (**A**) Changes in glucose levels in culture medium were determined using enzymatic assay. (**B**) Relative levels of GLUT1 mRNA in QB-treated cells compared to control (ctrl) were determined using RT-qPCR method. (**C**) Relative levels of GLUT1 protein in QB-treated cells compared to control (ctrl) quantified by LFQ analysis. (**D**) GLUT1 protein levels in QB-treated cells compared to control (ctrl) determined by immunoblot. (**E**) Relative levels of glycolytic enzymes, hexokinase (HK1), phosphofructokinase (PFK), glyceraldehyde 3-phosphate dehydrogenase (GAPDH), phosphoglycerate mutase (PGM1), and pyruvate kinase M (PKM) in QB-treated cells compared to control (ctrl) determined by LFQ analysis. (**F**) Fructose 6-phosphate levels in control (ctrl) and QB-treated cells (QB) determined by enzymatic assay. (**G**) Levels of O-GlcNAC modifications of individual proteins determined by immunoblot comparing treated (QB) and control (CTRL) cells. Data are shown as means from three independent experiments ± SEM. ^*^, significant differences with *P* ≤ 0.05. ^**^, significant differences with *P* ≤ 0.01. ^***^, significant differences with *P* ≤ 0.001. The images shown are representative of three independent experiments. In all experiments cells were treated with 2 µmol/L of QB.

To clarify possible changes in leukemia metabolism triggered by QB treatment, we focused on glycolytic pathway due to its prime role in cancer cell proliferation. As a first step, we quantified uptake of glucose during QB treatment that showed a decrease in the glucose levels in culture cell medium using enzymatic assay. We detected an insignificant increase in glucose uptake after QB treatment (Figure 2A). Considering that GLUT1 represents major glucose transporter in Jurkat cells, we tested the effects of QB on GLUT1 mRNA and protein levels using RT-qPCR, immunoblotting and LFQ analysis. We observed a significant decrease in GLUT1 mRNA levels and a slight increase in GLUT1 protein levels, which suggests potential stabilization of the GLUT1 protein on the plasma membrane (Figure 2B–2D).

### QB shows multiple effects on the glycolytic pathway functions

To characterize the effect of QB on the levels of individual enzymes of the glycolytic pathway, we analyzed LFQ data and quantified relative levels of individual glycolytic enzymes. We also quantified the levels of all glycolytic enzymes (except phosphoglucose isomerase and enolase). After QB treatment, the level of hexokinase (HK1) was not significantly increased, but the levels of phosphofructokinase (PFK), glyceraldehyde 3-phosphate dehydrogenase (GAPDH), phosphoglycerate mutase (PGM1), and pyruvate kinase M (PKM) were significantly elevated (Figure 2E). This result suggests an increased rate of glycolytic flux triggered by QB in leukemia cells. PFK catalyzes the irreversible conversion of fructose 6-phosphate to fructose 1,6-bisphosphate and represents one of the key regulatory glycolytic enzymes. Additionally, fructose 6-phosphate represents the substrate for glutamine--fructose 6-phosphate amidotransferase (GFAT), which acts as the rate-limiting enzyme in UDP-acetylglucosamine (UDP-GlcNAc) synthesis pathway that is indispensable for proper N- and O- protein glycosylation [26]. Hence, we quantified fructose 6-phosphate levels using enzymatic assay after QB treatment and observed a significant decrease (approx. 8-fold) in its level in cells treated with QB (Figure 2F). Since glucose uptake slightly increased after QB treatment, we presumed that low level of the fructose 6-phosphate was a result of increased PFK activity (GFAT represents enzyme with low-affinity to fructose 6-phosphate utilizing approx. 5% of the total glucose in the cell). PFK activity has been reported to be negatively regulated by ATP level, O-GlcNAcylation and low cytosolic pH (result of increased lactate concentration) and positively regulated by AMP levels (AMP diminished effect of ATP and pH) which were increased after QB treatment (Figure 1H and 1I) [27].

### QB treatment inhibits protein O-glycosylation in leukemic cells

Considering the effect on fructose 6-phosphate utilization, we focused on the determination of GFAT enzyme level. We detected an insignificant increase in levels of GFAT after QB treatment using LFQ analysis (data not shown). Hence, we monitored the effect of QB on protein glycosylation. Using immunoblotting, we observed nearly total abrogation of protein O-glycosylation after QB treatment (Figure 2G). These results suggest inhibition of protein O-glycosylation, probably due to low levels of fructose 6-phosphate, the first substrate in hexosamine synthetic pathway. Moreover, the absence of protein O-glycosylation possessed a strong effect on cellular oncogenic signaling, adhesion, proliferation and survival [28]. Several reports have highlighted the role of O-glycosylation in the stabilization of crucial oncogenic transcription factors (e.g. C-MYC) and pro-survival signaling pathways (e.g. PI3K) [29, 30]. It was also shown that O-GlcNAc modification of PFK molecule inhibited its enzyme activity, thereby, increasing glucose flux through the pentose pathway supporting cancer cells proliferation [26, 27].

### QB shows strong impact on 3PG-serine-glycine-5,10CH2-THF-10CHO-THF-formate biosynthetic pathway

Recently, novel metabolic pathway feeding mitochondrial respiratory complex I was decrypted. This pathway originated from serine-glycine synthesis pathway. In this pathway, glycolytic intermediate 3-phosphoglycerate feeds serine synthesis, which is converted in mitochondria to glycine by the activity of serine hydroxymethyltransferase (SHMT2) while producing 5,10-methylene-tetrahydrofolate (5,10-CH2-THF). Glycine can be subsequently utilized for protein and glutathione synthesis or can be transported out of the cell. Since a high concentration of glycine is considered highly cytotoxic, cellular level of glycine must be tightly regulated [31]. 5,10-CH2-THF is subsequently oxidized to 10-formyl-tetrahydrofolate (10-CHO-THF) by the activity of 5,10-methylene-tetrahydrofolate dehydrogenase (MTHFD2), which produces NADH that can serve as an electron donor to mitochondrial complex I. 10-formyl-tetrahydrofolate is then cleaved to THF and formate by the activity of reverse 10-formyl-tetrahydrofolate synthase generating one molecule of ATP from ADP [7].

**Figure 3:**
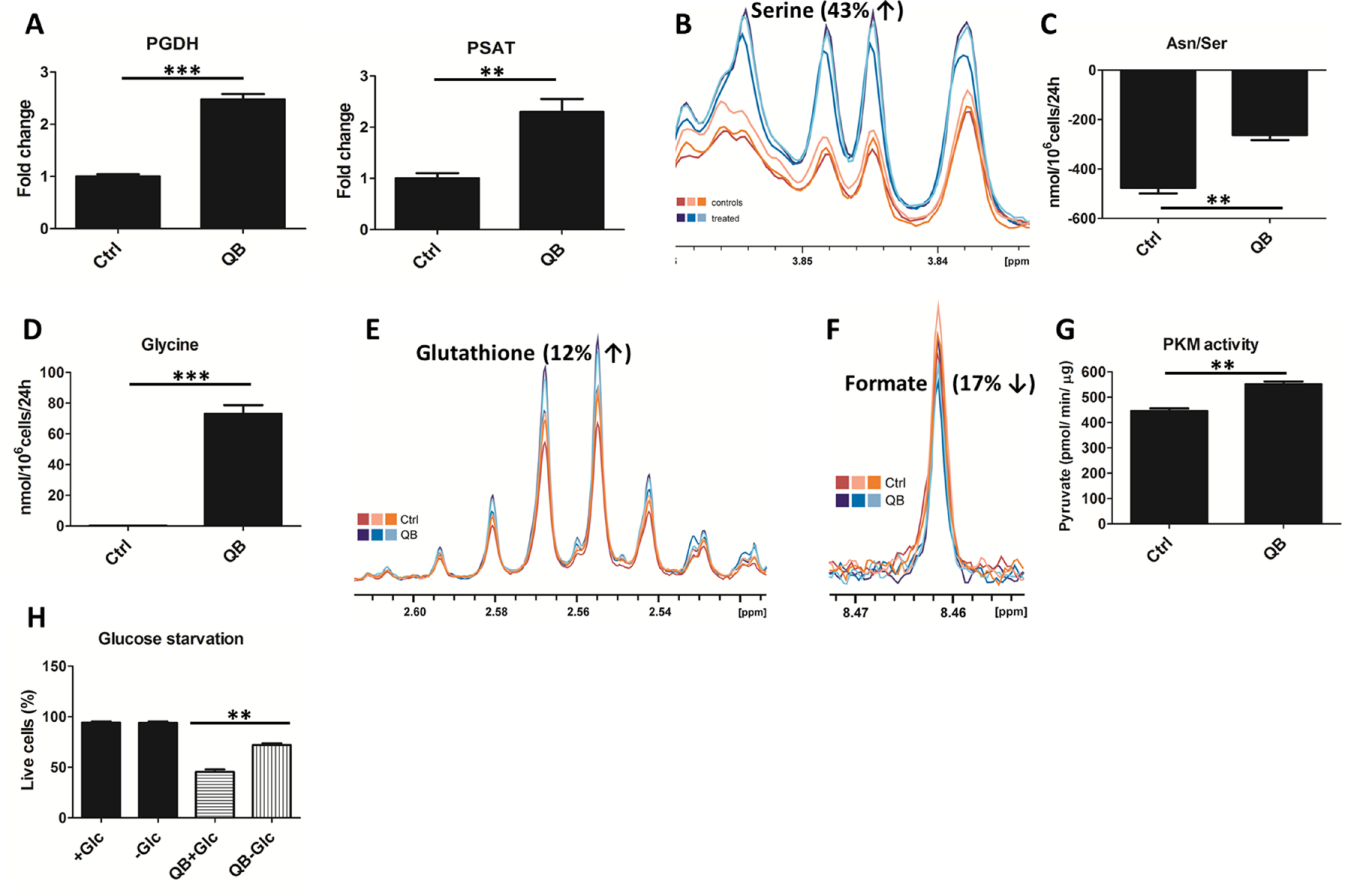
Effect of Quambalarine B (QB) on serine-glycine synthesis pathway (**A**) Relative levels of individual enzymes of serine synthesis pathway in control (ctrl) and QB-treated cells (QB) determined by LFQ analysis. (**B**) Intracellular levels of serine in control (orange lines) and QB-treated (blue lines) cells determined by NMR analysis. (**C**) Changes in Asn/Ser levels in culture medium after 24 h of control (ctrl) and QB-treated cells (QB) determined by HPLC analysis. (**D**) Changes in glycine levels in culture medium after 24 h of control (Ctrl) and QB-treated cells (QB) determined by HPLC analysis. (**E**) Intracellular levels of glutathione in control (orange lines) and QB-treated (blue lines) cells determined by NMR analysis. (**F**) Intracellular levels of formate in control (orange lines) and QB-treated (blue lines) cells determined by NMR analysis. (**G**) Pyruvate kinase M (PKM) activity in control (Ctrl) and QB-treated cells (QB) determined by enzymatic assay. (**H**) Percentage of dead cells in normal cultures and cultures under glucose starvation after QB treatment were determined by FACS analysis. Data are shown as means from three independent experiments ± SEM. ^*^, significant differences with *P* < 0.05. ^**^, significant differences with *P* < 0.01. ^***^, significant differences with *P* < 0.001. The images shown are representative of three independent experiments. In all experiments cells were treated with 2 µmol/L of QB

To uncover the effect of QB treatment on the glycine synthesis pathway, we quantified the levels of individual enzymes using LFQ analysis. We observed a significant increase in the levels of D-3-phosphoglycerate dehydrogenase (PGDH) (268% of non-treated control) and phosphoserine aminotransferase (PSAT) (223% of non-treated control), but no significant changes were observed in SHMT2 and MTHFD2 levels (data not shown) after QB treatment (Figure 3A). Then we determined the effect of QB on intracellular serine levels and uptake of serine from the culture medium using NMR and HPLC analysis. NMR analysis uncovered an increase in intracellular levels of serine (Figure 3B). Unfortunately, this result cannot be clearly confirmed by HPLC analysis due to the identical retention time of serine and asparagine. However, we observed a significant decrease in serine/asparagine uptake (approx. 50 % of non-treated control) in leukemic cells after QB treatment (Figure 3C). Using the same setup, we determined the levels of glycine and glycine uptake. We detected slight increase in the intracellular glycine levels after QB treatment using NMR analysis (data not shown), which probably reflected tight control of glycine concentration in cancer cells [31]. Surprisingly, we did not detect any significant uptake of glycine by control leukemic cells; however, we observed a significant increase in extracellular glycine levels during QB treatment using HPLC analysis, suggesting export of glycine out of the cell (Figure 3D). We also determined an increase in the glutathione (GSH) levels after QB treatment, which represented another possible route in the glycine metabolism [7] (Figure 3E). These results indicated that synthesis of glycine from glycolytic intermediate 3-phosphoglycerate can represent a major source of glycine in leukemic cells pointing to the exclusive role of glycolysis in leukemic cells proliferation. Then, we tracked the effect of QB on formate production employing NMR analysis and observed a decrease in the intracellular formate levels (Figure 3F). It is known that the rate of formate production depends on complex I activity since metformin and rotenone (inhibitors of complex I) inhibit formate production in several cancer cell lines [7, 32]. Mitochondria-derived formate represents an important intermediate in the cytosolic synthesis of purines or can be exported out of the cell. Hence, our observations provided another evidence of complex I blockade during QB treatment. Moreover, the high levels of formate and lactate production in non-treated cells suggest involvement of the folate pathway in mitochondrial metabolism.

### QB increases PKM activity in leukemic cells

PKM activity represents another important regulator of glycolytic flux. It is well known that cancer cells predominantly express PKM2 isoform, which possesses low enzymatic activity as a result of C-MYC oncogenic activity [4]. PKM2 expression triggers the accumulation of upper glycolytic intermediates, which can feed biosynthetic pathways derived from glycolysis [33]. On the other hand, serine represents one of the end-point glycolysis-derived metabolites and allosterically activates PKM2 activity [34]. We have previously demonstrated that QB treatment decreases C-MYC protein levels and PKM2 mRNA levels in several leukemic cells [24]. In this study, we also demonstrated an increase in the serine levels after QB treatment; hence, we tested PKM activity after QB treatment using enzymatic assay. We confirmed a significant increase in PKM activity triggered by QB treatment along with increased levels of intracellular pyruvate (Figures 3G and 1D). Several other compounds activating PKM activity in cancer cells were recently tested as potential anti-cancer drugs. These compounds impaired anabolic metabolism and inhibited cancer cell proliferation, demonstrating the important role of glycolysis-derived pathways in cancer cells proliferation and survival [35].

### Glucose starvation protects leukemic cells against cell death induced by QB

We tested the effect of glucose starvation to highlight the importance of glucose metabolism in QB anti-cancer activity. Cells were pre-incubated for 24 h in RPMI1640 medium without glucose supplemented with FBS followed by treatment with EC_50_ concentration of QB (8 µmol/L) for 24 h. Fractions of live/dead cells were determined using FACS analysis. Surprisingly, we detected significantly reduced fractions of death cells after glucose starvation against cells grown in standard cultivation medium (Figure 3H). This result suggests protecting effect of adaptation to glucose starvation against cell death induced by QB.

### QB treatment inhibits glutamine metabolism and aspartate synthesis in leukemia cells

**Figure 4:**
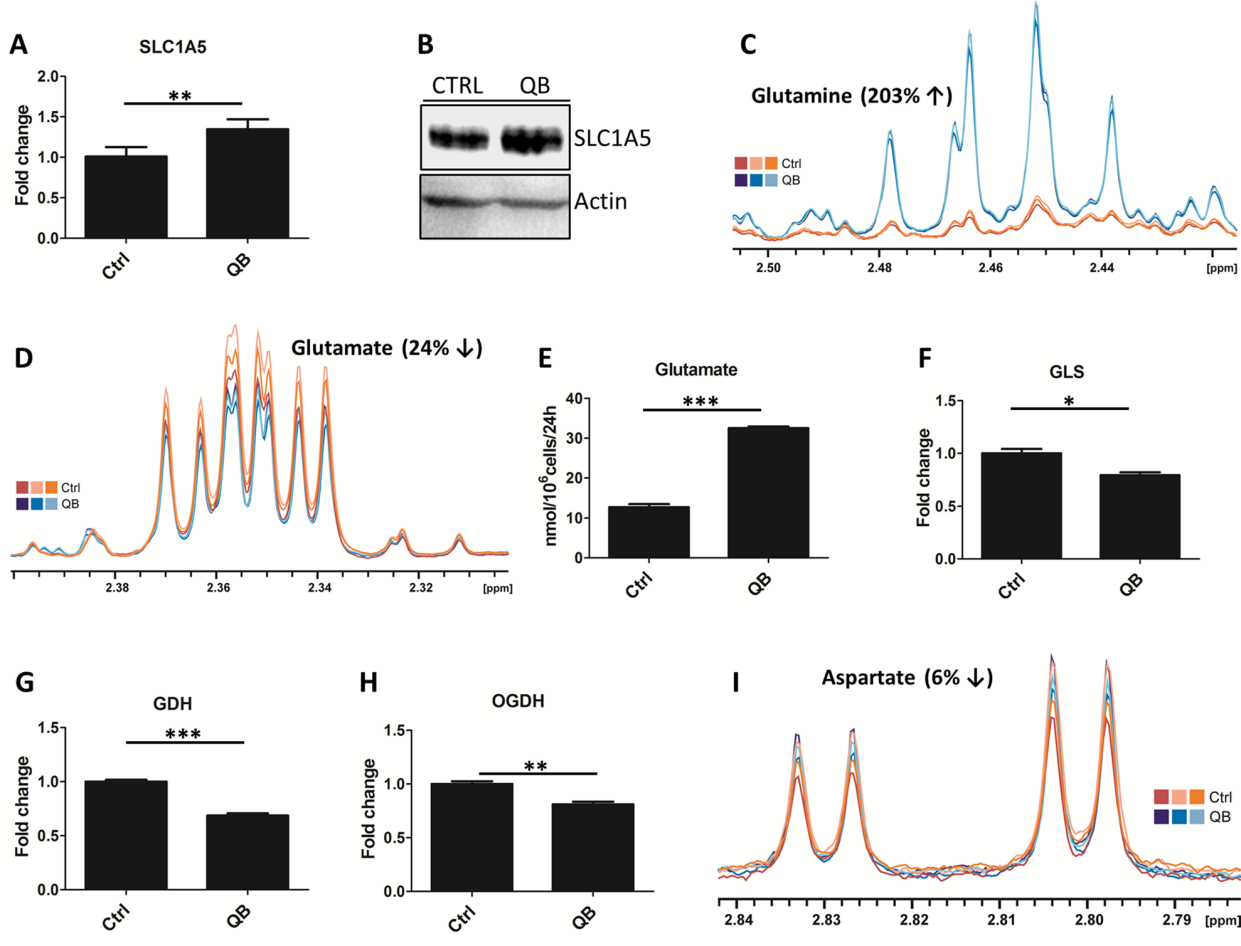
Quambalarine B (QB) deregulates glutamine metabolism in leukemic cells (**A**) Relative levels of glutamine transporter SLC1A5 in control (ctrl) and QB-treated (QB) cells determined by LFQ analysis. (**B**) Levels of SLC1A5 transporter in control (CTRL) and QB-treated cells visualized by immunoblot. (**C**) Intracellular levels of glutamine in control (orange lines) and QB-treated (blue lines) cells determined by NMR analysis. (**D**) Intracellular levels of glutamate in control (orange lines) and QB-treated (blue lines) cells determined by NMR analysis. (**E**) Changes in glutamate levels in the culture medium after 24 h of control (ctrl) and QB-treated (QB) cells determined by HPLC analysis. (**F**, **G**, **H**) Relative levels of glutamine metabolism enzymes: glutaminase (GLS), glutamate dehydrogenase (GDH), and alpha-ketoglutarate dehydrogenase (OGDH) in control (ctrl) and QB-treated (QB) cells determined by LFQ analysis. (**I**) Intracellular levels of aspartate in control (orange lines) and QB-treated (blue lines) cells determined by NMR analysis. Data are shown as means from three independent experiments ± SEM. ^*^, significant differences with *P* ≤ 0.05^**^, significant differences with *P* ≤ 0.01^***^, and significant differences with *P* ≤ 0.001. The images shown are representative of three independent experiments. In all experiments cells were treated with 2 µmol/L of QB.

We tested the effect of QB on glutamine metabolism and subsequent oxaloacetate and aspartate synthesis. We quantified the expression of SLC1A5 glutamine transporter using LFQ analysis and immunoblotting, and both the techniques detected a slight increase in SLC1A5 levels after QB treatment (Figure 4A, 4B). Using LFQ analysis we also observed increase by 1.3 and 1.5 folds for other potential glutamine transporters represented by SLC38A1 and SLC38A2, respectively (data not shown). Then, we monitored the glutamine uptake from the culture medium using HPLC analysis. Although glutamine and histidine shared similar retention time during HPLC analysis, we did not observe any significant change in glutamine/histidine transport (data not shown). We quantified the intracellular glutamine levels by NMR and observed its considerable increase after QB treatment, suggesting the inhibition of glutaminolysis and/or glutamine-dependent reactions (e.g. hexosamine and de novo purine synthesis) after QB treatment (Figure 4C). Next, we probed the effect of QB on glutamate transport and intracellular levels using NMR or HPLC, respectively. Using NMR, we observed a decrease in the intracellular glutamate levels, but on using HPLC, we observed an increase in the extracellular glutamate levels, suggesting an increased transport of glutamate out of the cell during QB treatment (Figure 4D and Figure 4E). Moreover, on using LFQ, we observed a significant decrease in enzymes involved in glutamine metabolism, such as glutaminase (GLS), GDH and alpha-ketoglutarate dehydrogenase (OGDH; Figure 4F, 4G and 4H). Unfortunately, we were unable to detect the levels of other metabolites of glutamine metabolism (from AKG to succinate), but we confirmed the increased levels of succinate (Figure 1C) after QB treatment as a result of complex II inhibition (confirmed also by oxygraph measurements). Finally, we determined the levels of aspartate after QB treatment and observed a slight decrease in aspartate levels in leukemic cells (Figure 4I). We also confirmed blockade of aspartate uptake from cell culture medium by Jurkat leukemic cells (data not shown). Inhibition of aspartate synthesis represented intriguing mechanism responsible for inhibition of cellular proliferation triggered by QB treatment.

### QB strongly deregulates levels of amino acids in leukemic cells

**Figure 5:**
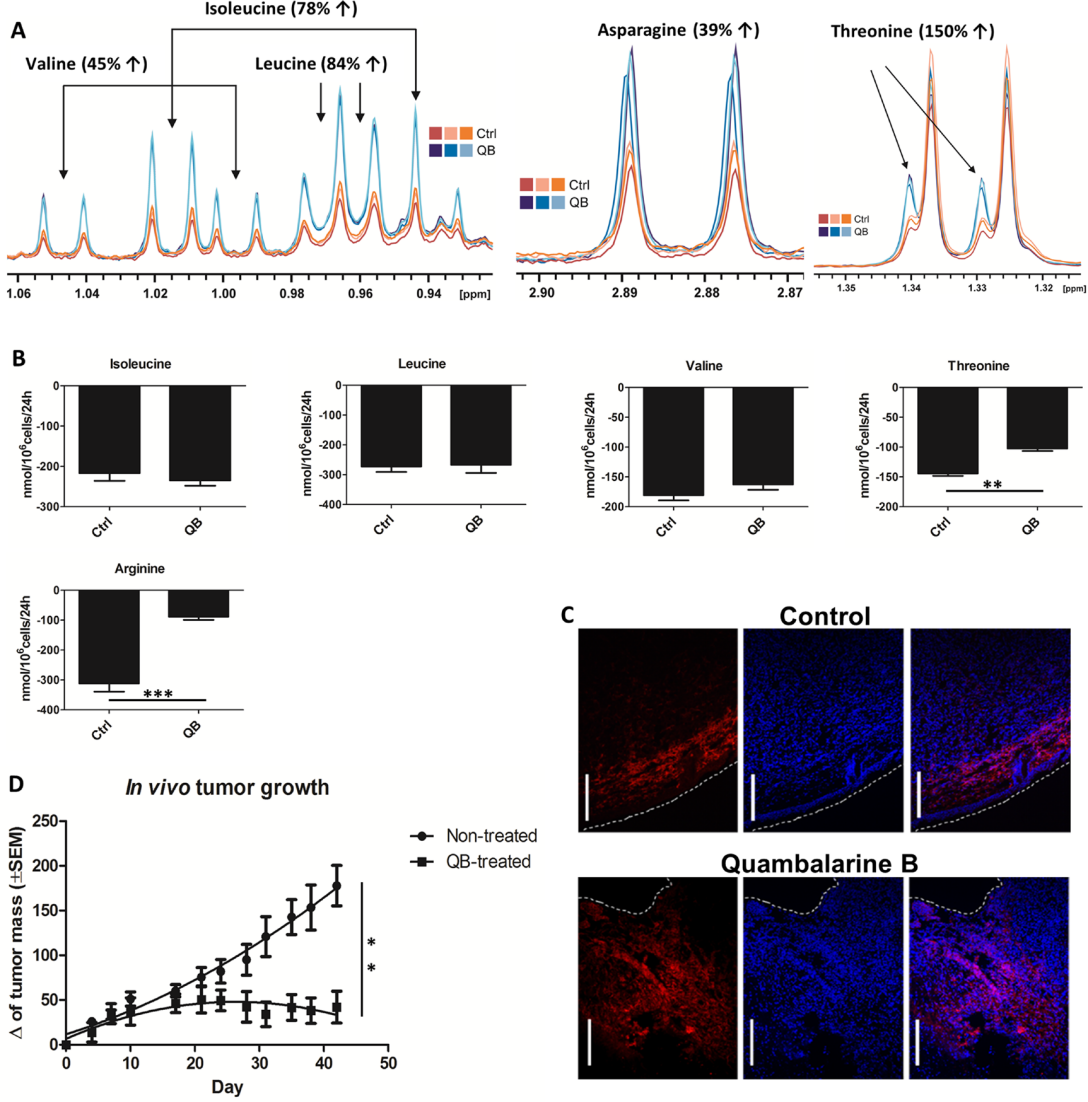
Quambalarine B (QB) increases levels of several AA in leukemic cells and inhibits tumor growth *in vivo* (**A**) Intracellular levels of valine, leucine, isoleucine, asparagine and threonine in control (orange lines) and QB-treated (blue lines) cells determined by NMR analysis. (**B**) Changes in individual AA levels in the culture medium of control (ctrl) and QB-treated (QB) cells after 24 h incubation determined by HPLC analysis. (**C**) Distribution of macrophages (F4/80+) in solid tumors derived from human breast cancer cell line MDA-MB-231 developed and treated with QB in the immunocompromised mouse model (blue - cell nuclei visualized by DAPI, red – mouse macrophages (F4/80+), dashed white line – tumor edge, white bar corresponds to 100 µm). (**D**) Effect of QB on tumor growth *in vivo*. Results from *in vivo* experiment represent mean ± SEM of value from one experiment (5 mice per group). All data are shown as means from three independent experiments ± SEM. ^*^, significant differences with *P* ≤ 0.05. ^**^, significant differences with *P* ≤ 0.01. ^***^, and significant differences with *P* ≤ 0.001.

Imbalance in AA metabolism represents an important hallmark of cancer cells with potential applications in cancer therapy [36, 37]. To explore the effect of QB on AA milieu in leukemic cells, we determined the uptake of individual AA using HPLC and quantified the intracellular levels of AA after 24 h of QB treatment using NMR analysis. We detected an increase in the intracellular levels of glycine, isoleucine, leucine, valine, asparagine, threonine, alanine, serine and glutamine and a decrease in the intracellular levels of glutamate and aspartate (Figures 1E, 3B, 4C, 4D, 4I, and 5A).

We observed no changes in isoleucine, leucine and valine uptake (essential AA), but we confirmed a significant decrease in threonine and arginine uptake. These results suggest the mechanism responsible for AA accumulation in Jurkat cells other than upregulated transport from the culture medium (Figure 5B). Suspect mechanisms can involve inhibition of protein translation and/or induction of autophagy by QB in leukemic cells. We identified the unexpected effect of QB on the uptake of alanine using HPLC. Control cells imported a significant amount of alanine from the culture medium. In contrary, the QB-treated cells exported a significant amount of alanine into medium (Figure 1F). Extracellular alanine could originate from glycolysis as a result of the pyruvate transamination, autophagy or inhibition of protein translation. Serine represented another nonessential AA, which was accumulated in the leukemic cells during QB treatment. Although serine retention time matched the asparagine characteristics during HPLC analysis, we observed a significant decrease in asparagine/serine uptake during QB treatment (Figure 3C). Based on these observations, we emphasized on increased synthesis of serine as a result of rewired glycolytic flux triggered by QB in Jurkat cells, autophagy or inhibition of protein synthesis. Glutamine represented the most accumulated AA in leukemic cells after QB treatment (Figure 4C). Although glutamine retention time matched to that of histidine retention time during HPLC analysis, no effect of QB treatment on glutamine/histidine transport was observed. Accumulation of glutamine seemed to originate from mitochondrial complex II blockade, nucleotides and hexosamine biosynthesis inhibition and/or autophagy together with protein translation stop. Since glutamine represents the most consumed AA by leukemic cells in a medium, we did not anticipate the involvement of glutamine synthesis in this process. Asparagine represents the second favorite AA, which has been eaten by leukemic cells from the culture medium, and the HPLC technique showed decreased asparagine/serine uptake by Jurkat cells during QB treatment (Figure 3C). Hence, increased intracellular levels of asparagine can be a result of autophagy and/or inhibition of protein translation as well. Glutamate represented AA, which can be generated in cells as a result of glutamine deamination catalyzed by GLS or glutamine-dependent enzymes. Surprisingly, leukemic cells did not uptake glutamate from the culture medium and rather export excess of glutamate into the medium. We observed increased production of glutamate by Jurkat cells into the medium after QB treatment, which can be a result of autophagy activation, protein translation inhibition or can reflect inhibition of TCA cycle that is fed mainly through glutaminolysis (Figure 4E). A similar effect was observed for glycine during QB treatment. Control cells did not transport any glycine from the culture medium, but QB-treated cells produced a significant amount of glycine into the medium. Finally, aspartate represented the only AA, which was generally decreased in Jurkat cells after QB treatment, and we detected neither uptake nor export of aspartate by leukemic cells. Since the synthesis of aspartate by leukemic cells seemed to be blocked by QB, we presumed autophagy and/or inhibition of protein synthesis as the mechanisms buffering aspartate levels under QB pressure.

### QB maintains its anti-tumor activity in *in vivo* mouse xenograft model

For the *in vivo* test of the anti-tumor activity of QB, the cell line MDA-MB-231 (human breast carcinoma) was used. Cells were introduced subcutaneously, and the size of the developing tumors was measured. The result clearly showed inhibition of the tumor growth on a statistically sufficient set of animals. At the end of the experiment, tumors were removed and subjected to immunohistological study comparing the control vs. treated tumors. A clear distinction was observed in the pattern and quantity of tumor infiltrating macrophages (F4/80+ cells). In control tumors, the macrophages were concentrated in a sharp line close to the tumor capsule. In tumors removed from the mouse treated with QB, macrophages infiltration was massive and reached much deeper into the tumor. Clear distinction was also found in the histological composition of the cancer edge. Control tumors were enveloped by E-cadherin highly positive stratified mouse epithelia, whereas the surface of tumors from treated mice was “eroded” with much less epithelial cover (data not shown). Data from *in vivo* mouse xenograft model clearly supported the notion that QB maintained its anti-tumor activity in *in vivo* conditions (Figure 5C and 5D).

## DISCUSSION

We present here the proof of principle that metabolo-proteomic approach could be useful for deciphering molecular mechanisms underlying novel drugs action. Molecular simulations pointed out mitochondrial respiratory complexes I and II as potential targets of QB in Jurkat cells together with ubiquinone-competitive properties of QB. Mitochondrial respiratory complexes were recently showed as intriguing targets for cancer and diabetes therapy [38–40]. As the activity of these complexes is tightly linked to other metabolic pathways, we tracked alterations in these pathways during treatment of leukemic cells with IC_50_ concentration of QB as determined by MTS assay. The treatment showed the inhibitory effect on cellular proliferation, did not induce cell death and led to the rearrangement of metabolism of Jurkat cells [24]. Inhibition of ETC by QB led to increased levels of intracellular AMP, which inhibited anabolic pathways crucial for leukemic cells proliferation. AMP is a well-known stimulator of PFK activity, which preferentially feeds downstream parts of glycolysis to support anaplerotic substrates for mitochondrial metabolism. Enhanced PFK activity results in depletion of upstream glycolytic intermediates crucial for pentose and hexosamine biosynthetic pathways. As a potential result of high AMP levels, we confirmed a decrease in O-glycosylation of proteins, which was represented as an important regulator of stability of several oncogenic factors such as C-MYC. Consistent with this hypothesis, C-MYC levels were significantly downregulated during QB treatment [24]. Hence, inhibition of hexosamine pathway and protein O-glycosylation provided a new possibility to regulate leukemia cells proliferation [41]. In agreement with these findings, we detected increased levels of glycolysis-derived intermediates feeding mitochondrial metabolism represented by pyruvate, succinate, and serine. Inhibition of mitochondrial complexes I and II by QB slowed down the utilization of electron donors by ETC followed by accumulation, conversion, and export of such metabolites out of the cell. These reactions involved conversions of serine to glycine and pyruvate to alanine together with the inhibition of pyruvate to lactate conversion. Glycine can be metabolized to toxic products, amino acetate and methylglyoxal, a reactive aldehyde, which covalently modifies AA residues in proteins [31]. Export of glycine out of cell seemed to represent an important protective mechanism in leukemia cells during ETC blockade. This fact highlighted glycine export machinery as a novel target for leukemia therapy. On the other hand, alanine acted as a potent inhibitor of PKM activity and pyruvate synthesis resulting in accumulation of upstream glycolytic intermediates [33]. Hence, metabolism and transport of alanine represented other potential therapeutic target. Next, serine acted as an allosteric activator of PKM activity, which was increased during QB treatment [34]. Inhibition of serine to glycine conversion can increase lactate levels and toxicity in cancer cells due to PKM activity stimulation as well as inhibit folate-driven ETC activity and purine biosynthesis. Contrary to our findings, recent reports claimed the inhibitory effect of naphthoquinonic compound shikonin on PKM activity in cancer cells [42]. Since shikonin possesses unsubstituted C2 position in its structure, we presume covalent binding of shikonin to regulatory cysteines in PKM molecule as a potential mechanism responsible for shikonin-driven inhibition of PKM [43]. Consistent with this assumption, we observed activation of ERK kinase by shikonin in Jurkat cells as a potential result of regulatory phosphatases inhibition (Supplementary Figure 2) [22].

We have also detected increased levels and export of several AA, except aspartate, in Jurkat cells during QB treatment. Increased levels of AA can originate from de novo synthesis as well as autophagy or inhibition of protein translation. Aspartate represents one of the key building blocks for nucleotide and protein synthesis. Jurkat cells showed extremely low uptake of aspartate from culture medium and were strongly dependent on aspartate synthesis from oxaloacetate [9]. We suggest that when oxaloacetate synthesis became inhibited by QB through complex II blockade, the levels of aspartate were transiently supported by autophagy followed by proliferation arrest. Increased levels of intracellular glutamine and extracellular glutamate also suggest inhibition of mitochondrial metabolism during QB treatment. These results demonstrated inhibition of aspartate synthesis as a promising therapeutic target for leukemia therapy. Moreover, inhibition of complex I activity together with the effect of QB on glycolytic flux rendered QB as a potential anti-diabetic drug. Several well-known anti-diabetic drugs act as inhibitors of ETC and decrease glucose levels in patient’s serum [44]. Since QB anti-cancer activity was confirmed also during *in vivo* studies, the practical therapeutic potential of QB will be extensively tested in the future.

## MATERIALS AND METHODS

### Cell line, culture conditions and reagents

The T-cell lymphoma-derived Jurkat cell line, clone E6.1, was obtained from the ATCC collection (ATCC, Manassas, VA, USA) and cultured at 37°C and 5% CO_2_ in the RPMI1640 medium supplemented with L-glutamine (Lonza Group, Ltd., Basel, Switzerland), 10% fetal bovine serum (FBS) (Gibco, USA), 100 U/mL penicillin, and 100 µg/mL streptomycin (PAA Laboratories, Austria). Cells were treated with 2 µmol/L concentration of QB. Unless otherwise stated, cells were seeded at a density of 2.5 × 10^5^ cells/mL in 15 mL of the RPMI1640 medium, cultured overnight in 75 cm^2^ cell culture flasks under standard cultivation conditions, treated with QB for 24 h and together with control cells immediately counted, harvested, washed with PBS and stored in deep freezer for subsequent analysis. For all experiments, QB stock solution was used (25 mmol/L in DMSO, kept in −20°C).

### RNA isolation, reverse transcription and RT-qPCR analysis

Cells were pelleted (400 × g, 4 min), washed with PBS buffer and lysed in a cell lysis buffer (Aurum RNA Isolation Kit, Bio-Rad, USA). Cell lysate was processed according to the manufacturer’s protocol, including an in-column DNase treatment. The purified RNA was quantified and tested for the presence of contaminants with a NanoDrop spectrophotometer (Thermo Fisher Scientific, USA). Reverse transcription of 700 ng of each purified RNA sample was performed using the M-MulV Taq RT-PCR Kit (New England Biolabs, USA). For individual RT-qPCR reaction mixtures, 2 µL of cDNA from the reverse transcription protocol were used. Individual reactions were run in triplicate using a CFX96 Real-Time PCR System (Bio-Rad, USA) in final volumes of 25 µL. Specific primers and the SsoFast™ EvaGreen Supermix (Bio-Rad, USA) were used for amplification and fluorescent detection of PCR products, respectively. The relative quantities of cDNA from treated and control cells were calculated by the Livak and Schmittgen 2^-∆∆Ct^ method [45]. P0 was used as a reference gene. Following primers were used for specific gene amplification: GLUT1-Forward TCGT CGTC GGCA TCCT CATC, GLUT1-Reverse CGGTTGATGAGCAGGAAGCG; P0-Forward TCGA CAAT GGCA GCAT CTAC, P0-Reverse ATCC GTCT CCAC AGAC AAGG.

### SDS electrophoresis and immunoblotting

For whole cell lysate analyses, cells were washed with PBS supplemented with a Phosphatase Inhibitor Cocktail (Active Motif, Belgium), lysed using the RIPA buffer (1% NP-40, 150 mmol/L NaCl, 0.5% NaDOC, 0.1% SDS and 50 mmol/L Tris-HCl pH 8) supplemented with Complete Protease Inhibitor Cocktail Tablets (F. Hoffmann-La Roche Ltd., Switzerland), and incubated for 30 min on ice. The cell lysate was cleared via centrifugation (14 000 × g, 10 min, 4°C). Bicinchoninic acid (BCA) assay was used to determine total protein concentration in the cell lysates (BCA Protein Assay Kit, Thermo Fisher Scientific, USA). Nuclear and cytosolic fractions were isolated from Jurkat cells using Nuclear Extract Kits (Active Motif, Belgium). SDS PAGE gels were loaded with 40 µg of protein per lane from individual samples. A Protean III apparatus (Bio-Rad, USA) with a constant voltage of 100 V was used to run the SDS PAGE protein samples. Separated proteins were blotted onto a nitrocellulose membrane (GE Healthcare, USA) using a Trans-Blot™ SD Semi-Dry apparatus (Bio-Rad, USA). Protein blots were blocked for 1 h in TBS supplemented with 5% milk (Bio-Rad, USA) and 0.05% Tween-20 (Sigma-Aldrich). Membranes were then washed with TBS containing 0.05% Tween-20 and incubated with the respective primary and secondary antibodies, according to manufacturer’s protocols. The following antibodies were used for immunostaining: anti-GLUT1 and anti-SLC1A5 rabbit primary antibodies (Abcam, UK). Secondary antibody against rabbit IgG were purchased from Santa Cruz Biotechnology.

### Effect of glucose deprivation on response of Jurkat cell line to QB

Jurkat cells were seeded in 24-well plate at a density 2 × 10^5^ cells/mL and cultured in final volume of 1 mL in complete RPMI1640 medium or medium lacking glucose. After 24 h incubation with EC_50_ of QB, fractions of live, apoptotic and dead cells were determined using Hoechst 33258 staining by flow cytometry measurement (FACS LSRII instrument, BD Biosciences, San Jose, CA, USA). Cells cultured in RPMI1640 with DMSO only were used as a control. Data analysis was performed using the FlowLogic 600.A software (Inivai Technologies, Australia).

### Metabolite extraction

The extraction was performed using 10^7^ cells per sample according to Gottschalk et al. protocol [46]. Cells were incubated according to standard protocol, counted, pelleted into a 1.5 mL vial and washed with PBS. Then 600 µL of a 2:1 (v/v) ratio of ice-cold methanol/chloroform was added, the pellets were re-suspended using a vortex mixer and incubated for 10 min on a rotator. Then 600 µL of ice-cold 1:1 (v/v) chloroform/water was added, mixed using a vortex and sonicated in the sonicating bath for 10 min. Samples were then centrifuged at 13 000 rpm for 5 min. The top layer was removed to a new vial taking care not to disturb the pelleted debris. The solvent was evaporated using a centrifugal concentrator and samples were frozen (−80° C) for later analysis.

### NMR metabolomics

The dried hydrophilic cell extracts were re-dissolved in a mixture of 450 μL D_2_O and 50 μL deuterated potassium buffer (1.5 mol/L KH2PO4 in D_2_O containing 2 mmol/L NaN3 and 0.1% trimethylsilyl propionic acid (TSP) as an internal standard; pH= 7.4) and transferred to a 5-mm NMR tube. The NMR spectra were recorded on a 600 MHz Bruker Avance III spectrometer (Bruker BioSpin, Rheinstetten, Germany) equipped with a 5-mm TCI cryogenic probe head.

All NMR experiments were performed at 298 K. Standard 1H NMR spectra were acquired using nuclear Overhauser enhancement spectroscopy (1D-NOESY) pulse sequence with following acquisition parameters: number of scans (NS) = 512, 64 k of data points (TD), spectral width (SW) of 20 ppm, relaxation delay (D1) of 4 s, a mixing time (D8) of 10 ms. The resonances of water were suppressed by presaturation during relaxation delay and mixing time. J-resolved experiment with presaturation (NS = 16, SW = 16 ppm, TD = 16 k, number of increments = 40, SW = 78.125 Hz in the indirect dimension, and relaxation delay = 2 s) was performed to facilitate the identification of metabolites. Additionally, 2D correlation spectroscopy (COSY) and heteronuclear single-quantum correlation experiments (HSQC) were measured for selected samples using standard manufacturer’s software Topspin 3.2 (Bruker BioSpin, Rheinstetten, Germany).

The acquired free induction decays (FIDs) were multiplied by an exponential window function (LB = 0.3 Hz). The spectra were automatically phased, baseline corrected and referenced to TSP (0.0 ppm). The processed 1D-NOESY spectra (0.10 –10.0 ppm) were uniformly binned and normalized to the total spectral area in Amix 3.9.14 software (Bruker Biospin, Rheinstetten, Germany) using the 0.01 binning step. The intensity of the respective signal, i.e. metabolite, was then determined as the sum of the corresponding 0.01-ppm bins to cover the whole signal width. The changes of some metabolites (glycine, threonine) were due to overlap with signals of other metabolites determined using Chenomx NMR Suite 7.6 (Chenomx Inc., Edmonton, AB, Canada). Regions corresponding to water (4.70–4.90 ppm) were removed prior to binning process. The peak assignment was performed also using Chenomx NMR Suite 7.6, and the HMDB database and published assignments [47]. The metabolite identification was supported by J-resolved and COSY experiments and confirmed using 2D NMR experiments.

### HPLC analysis of amino acids uptake from culture medium

Cells were seeded in six-well plates at concentration 10^6^ cells/mL and treated with QB. Cell were then counted using Bürker counting chamber, pelleted by centrifugation (400 x g, 4 min), medium was removed and centrifuged (12000 x g, 10 min) to remove cell debris. Medium incubated in six-well plates without cells was used as a control. High performance liquid chromatography analysis of amino acids was performed using Waters AccQ-Tag Chemistry Package (WAT052875) on two pump Beckman Coulter Gold chromatograph with Merck-Hitachi F-1080 fluorescence detector (ex.250 nm, em. 395 nm). Data were collected and evaluated with DataApex CSW32 chromatography software.

### Determination of glucose uptake from medium

Cells were seeded in six-well plates at concentration 5 × 10^5^ cells/mL and treated with QB or corresponding DMSO. After incubation, cells were counted using Bürker counting chamber, pelleted (400 g, 4 min, RT) and supernatant was centrifuged again (14 000 g, 10 min, RT) to eliminate cell debris. Final supernatant and control medium were diluted 50 times by adding dH_2_O, glucose concentration was determined using Glucose (GO) Assay Kit (Sigma-Aldrich, USA) according to the manufacturer protocol.

### Determination of cellular fructose-6-phosphate levels

To determine the fructose 6-phosphate (F6P) concentration, the PicoProbeTM Fructose 6-Phosphate Fluorometric Assay Kit (BioVision, USA) was used. Cells were cultivated in 50 ml of medium in 150 cm^2^ flasks at concentration 20 × 10^6^ and treated with QB. To deproteinize the samples, cells were lyzed in 200 µl of dH_2_O and then centrifuged (14 000 g, 10 min, RT). Supernatants were transferred to the centrifugal filter units with molecular weight cut-off of 10 kDa (Merck Millipore, Germany) and F6P was detected in the acquired filtrates according to the manufacturer protocol.

### Determination of lactate production

Lactate production was determined using Lactate Assay Kit (Sigma-Aldrich, USA). Cells were seeded at concentration 2.5 × 10^5^ cells/mL and treated with QB or DMSO (control samples) for 24 h. After treatment cells were transferred to six-well plate at concentration of 10^6^ cells/mL and after 8 h centrifuged (400 g, 4 min and 12 000 g, 10 min RT). Supernatant was analyzed for the lactate concentration according to manufacturer protocol.

### Determination of PKM kinetics

PKM kinetics was determined using Pyruvate Kinase Activity Kit (BioVision, USA). Cells were seeded at concentration 2.5 × 10^5^ cells/mL and directly treated with QB or DMSO. Cells were then lysed using Lysis buffer (BioVision, USA) and protein concentration was determined using BCA assay. One µg of extracted proteins from QB-treated and control cells was used for PKM activity determination in fluorescence mode.

### Determination of pyruvate cellular levels

Cells were cultivated and treated according to standard protocol. Pyruvate concentrations were determined in equal amounts of cells using Pyruvate Kinase Activity Kit (BioVision, USA) according to the manufacturer protocol.

### Label-free quantification analysis (LFQ)

To determine changes in Jurkat cell proteome caused by QB treatment, label-free quantification analysis was performed. We rely on sodium deoxycholate (SDC) and phase-transfer surfactant (PTS) method [48]. Cell pellets (1–2 × 10^6^ cells) were lyzed in 100 mmol/L triethylammonium bicarbonate (TEAB) containing 2% SDC (50 µl of buffer/1 million of cells). To eliminate DNA, cell lyzates were sonicated (10 min or until clear) or treated with benzonase (50 U/mL, 30 min on ice). Thereafter, the samples were boiled for 5 min and centrifuged (14 000 g, 5 min, RT). Bicinchoninic assay (Thermo Fisher Scientific, USA) was used to determine protein concentration in the supernatants, the amount of 20 µg of proteins was sufficient for a mass spectrometric measurement. Following cysteines reduction by 5 mmol/L triscarboxyethyl phosphine (TCEP; 30 min, 60°C), cysteines were alkylated using methyl methanethiosulfonate (MMTS) of the final concentration of 10 mmol/L (10 min, RT). The next step involved enzymatic digestion by trypsin in the ratio 1:20 (w/w) trypsin:proteins (overnight, 37°C), then the reaction was stopped by adding trifluoracetic acid (TFA) to the final concentration of 0.5 %. Finally, SDC was removed from samples using ethylacetate (PTS method) and peptides were desalted (peptide MicroTrap column, C18 phase, Michrom Bioresources, USA).

### nanoLC-MS^2^ analysis

Nano reversed phase columns (EASY-Spray column, 50 cm × 75 µm ID, PepMap C18, 2 µm particles, 100 Å pore size) were used for LC/MS analysis. Mobile phase buffer A was composed of water, 2% acetonitrile and 0.1% formic acid. Mobile phase B was composed of 80% acetonitrile and 0.1% formic acid. Samples were loaded onto the trap column (Acclaim PepMap300, C18, 5 µm, 300 Å Wide Pore, 300 µm × 5 mm, 5 Cartridges) for 4 min at 15 μl/min. Loading buffer was composed of water, 2% acetonitrile and 0.1% trifluoroacetic acid. Peptides were eluted with mobile phase B gradient (2% to 40%) in 120 min. Eluted peptide cations were converted to gas-phase ions by electrospray ionization and analyzed on a Thermo Orbitrap Fusion (Q-OT- qIT, Thermo Scientific, USA). Survey scans of peptide precursors from 400 to 1600 m/z were performed at 120K resolution (at 200 m/z) with a 5 × 10^5^ ion count target. Tandem MS was performed by isolation at 1.5 Th with the quadrupole, HCD fragmentation with normalized collision energy of 30, and rapid scan MS analysis in the ion trap. The MS^2^ ion count target was set to 104 and the max injection time was 35 ms. Only those precursors with charge state 2–6 were sampled for MS^2^. The dynamic exclusion duration was set to 45 s with a 10 ppm tolerance around the selected precursor and its isotopes. Monoisotopic precursor selection was turned on. The instrument was run in top speed mode with 2s cycles.

### LFQ data analysis

MaxQuant 1.5.3.8 peak picking software and Andromeda search engine were used in label-free experiments. Followed parameters were set during measurement: enzyme specificity considered- Trypsin/P, number of missed cleavages permitted – 2, fixed modification(s) – S-Methyl-L-cysteine sulphoxide (C), variable modification – Oxidation (M), Acetyl (Protein N-term), mass tolerance for precursor ions – 4. 5 ppm and mass tolerance for fragment ions – 0. 5 Da. False discovery rate (FDR) was set as follows: peptide spectrum match FDR – 1%, Protein FDR – 1% and min. score for modified peptides – 40. Human protein database downloaded from uniprot.org on 9/15/2015 with 147 933 entries was used for individual proteins annotation. The “match between runs” feature of MaxQuant was used to transfer identifications to other LC-MS/MS runs based on their masses and retention time (maximum deviation 0.7 min). This setup was also used in quantification experiments. Quantifications were performed with the label-free algorithms described recently [49]. All MS experiments were done in biological triplicates and measured in technical duplicates.

### Mitochondria isolation procedure

Liver was dissected out from sacrificed rat (Wistar, Animal facility of Institute of physiology CAS) and put in ice-cold isolation medium (0.25 mol/L saccharose, 20 mmol/L TRIS, 1 mmol/L EDTA, pH 7.4). Approximately 5 g of the tissue was taken for isolation, minced with scissors in ice-cold isolation medium, washed and then re-suspended with ice-cold isolation medium and homogenized with Potter-Elvehjem homogenizer (glass-teflon) by six strokes at 800 rpm using a digital overhead stirrer (VELP Scientifica). Homogenate was transferred into two 50 ml Falcon tubes and centrifuged at 800 g, 10 min, 4°C, supernatant was centrifuged (10 000 g, 10 min, 4°C), washed with ice-cold isolation medium, centrifuged (10 000 g, 10 min, 4°C), washed with ice-cold isolation medium, transferred into 2 tubes and centrifuged (10 000 g, 10 min, 4°C). Finally, purified mitochondria were re-suspended in ice-cold isolation medium, kept on ice and immediately used for high-resolution respirometry measurement by OROBOROS Oxygraph-2k instrument (Innsbruck, Austria). Aliquots were stored at −20°C for determination of protein concentration.

### Mitochondrial respiration

Respiratory oxygen flux was measured by OROBOROS Oxygraph-2k instrument in real-time at 30°C. Respiration stimulation was determined using substrates for individual mitochondrial complexes I and II. Isolated mitochondria were added into respiratory medium (80 mmol/L KCl, 10 mmol/L Tris, 3 mmol/L MgCl2, 1 mmol/L EDTA, 5 mmol/L KH2PO4, pH 7.4) in respective chambers. First, glutamate (10 mmol/L) and malate (2 mmol/L) were added (substrates for mitochondrial complex I) and cytochrome C (10 µmol/L) to test outer mitochondrial membrane integrity. Addition of ADP (2 mmol/L) shows inner membrane integrity and coupling of oxidation and phosphorylation. Addition of succinate (10 mmol/L) indicates mitochondrial complex II function. Combination of ascorbate (2 mmol/L) and TMPD (0.5 mmol/L) is substrate for cytochrome C oxidase. Final inhibition with KCN (1 mmol/L) excludes nonspecific oxygen consumption. Index of respiratory control was determined as a ratio between respiration at state III (+ADP) and state IV (-ADP). Oroboros software Datlab 5 version 5.1.0.20 (Oroboros Instruments Corp, Innsbruck, Austria) was used for calculating oxygen uptake rates and for presenting the oxygraphic curve. Oxygen uptake in isolated mitochondria is expressed as pmol/s/mg protein [50]. Presented data are subject of three independent experiments performed in technical duplicates.

### Mouse model

The cell line MDA-MB-231 (Human breast carcinoma, Leibniz Institute DSMZ-German Collection of Microorganisms and Cell Cultures, Braunschweig, Germany) was used for in *in vivo* experiments. Cells were cultured under following conditions: 37°C, 5% CO_2_, Dulbecco's Modified Eagle's Medium (DMEM) with 10% FBS, 2 mmol/L L-glutamine, and non-essential amino acids 100× (all Sigma Aldrich, USA).Outbred mouse females (nu/nu strain Foxn-1) were used for xenograft experiment, with a body weight 20–22 g (AnLab. Ltd., Czech Republic; bred in Centre for Experimental Biomodels, Charles University, Czech Republic). Mice were placed in IVC cages with radiation-sterilized bedding SAWI- Research Bedding (AnLab, Czech Republic) fed with radiation sterilized diet ssniff (AnLab Ltd., Czech Republic) and autoclaved water ad libitum. A lighting regimen was 12 h light/12 h darkness, temperature 23 °C and humidity 50–60%.Mice were transplanted with MDA-MB-231cell line (1 × 10^7^ tumor cells/mouse in a 100 µl volume together with 50 µl of matrigel – Sigma, USA) subcutaneously to the right flank. The application of the test compound was initiated 7 days later. QB was applied by injection into eye in 8 separate dosages (0. 2 mg in DMSO per animal) twice a week. During experiment, sizes of tumors were measured with caliper. The mice were euthanized by overdosing the anesthetics at the end of the experiment tumors were removed and subjected to further analysis.

All animal studies were carried out according to the experimental project approved by Ministry of Education, Youth and Sport of the Czech Republic.

### Histology

Dissected tumours were fixed in 3.8% paraformaldehyde in PBS, washed in PBS and transferred in 30% saccharose in PBS. Tumours were embedded into mounting medium (Cryomount) on dry ice and stored at −80°C. Cryosections (12 µm) were prepared using cryostat Leica CM3050 S and stored at −80°C. Sections were permeabilized by 0.5% Triton X-100 in PBS, blocked by 1% BSA in PBS and stained with rat anti-mouse F4/80 primary antibody (EXBIO, Prague, Czech Republic), followed by staining with goat anti-rat IgG (H+L) secondary antibody Alexa Fluor 594 conjugate (Thermo Fisher Scientific, USA). Staining of nuclei and sample mounting was performed by Fluoroshield with DAPI (Sigma-Aldrich). Visualisation was performed using an inverted microscope (Olympus IX71) using 20x objective. Acquisition and data processing was performed using DP Controller 2.2.1.227 software.

### Molecular modeling

Crystal structures of yeast mitochondrial complex I (CI) from Yarrowia lipolytica (PDB ID 4wz7) and porcine mitochondrial complex II (CII) (PDB ID 1zoy) were used for modeling. The program 3V was used to identify internal cavities connecting the iron-sulfur clusters with the predicted ubiquinone binding cavity in the CI crystal structure [51]. The geometry of ubiquinone and QB was optimized using DFT-D method with TPSS functional and TZVP basis set [52, 53]. The effect of water solvation was treated implicitly using COSMO with ε = 78.4. All the optimizations were performed in the TurboMole suite of programs. The Python Molecular Viewer (PMV 1.5.6 rc3) was used to set the docking parameters [54]. AutoDock Vina version 1.1.2 was used for ubiquinone and QB docking to CI and CII structures [55]. For the CI structure, the ligands were allowed to sample docking poses in a 60 × 60 × 60 Å box centered around the predicted ubiquinone binding cavity, covering large portion of the lower part of the peripheral arm (Q module) and the transmembrane PP module of the membrane arm. For the CII structure the ligands sampled a box of 30 × 30 × 30 Å around the known crystal position of the ubiquinone moiety.

## SUPPLEMENTARY MATERIALS FIGURE


