# Supplementary material for: Reprogramming of leukemic cell metabolism through the naphthoquinonic compound Quambalarine B
Source: Oncotarget. 2017 Oct 7;8(61):103137–53. doi: 10.18632/oncotarget.21663 (PMC5732718; doi:10.18632/oncotarget.21663)
Supplement: Supplementary file 1 [file oncotarget-08-103137-s001.pdf]

Reprogramming of leukemic cell metabolism through the naphthoquinonic compound Quambalarine B

SUPPLEMENTARY MATERIALS

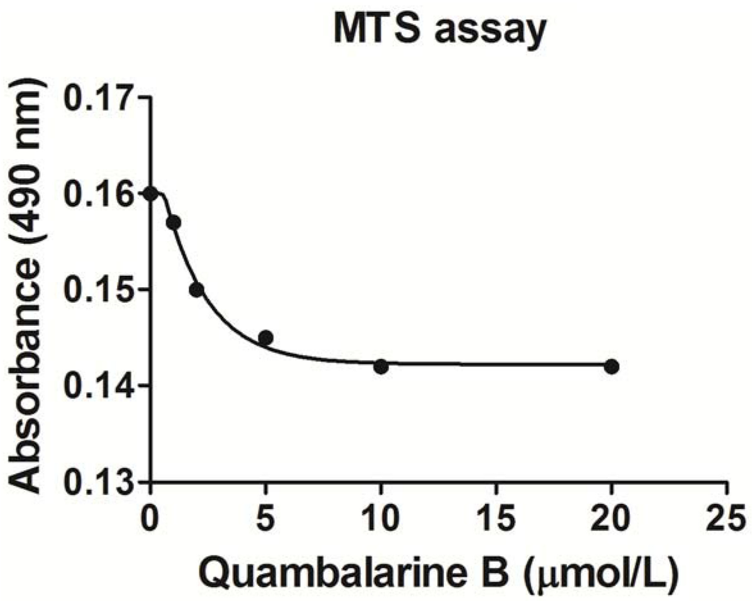

Supplementary Figure 1: Determination of Inhibitory Constant (IC_{50}) of Quambalarine B (QB) toward cellular oxidoreductases in Jurkat cell line using MTS assay.

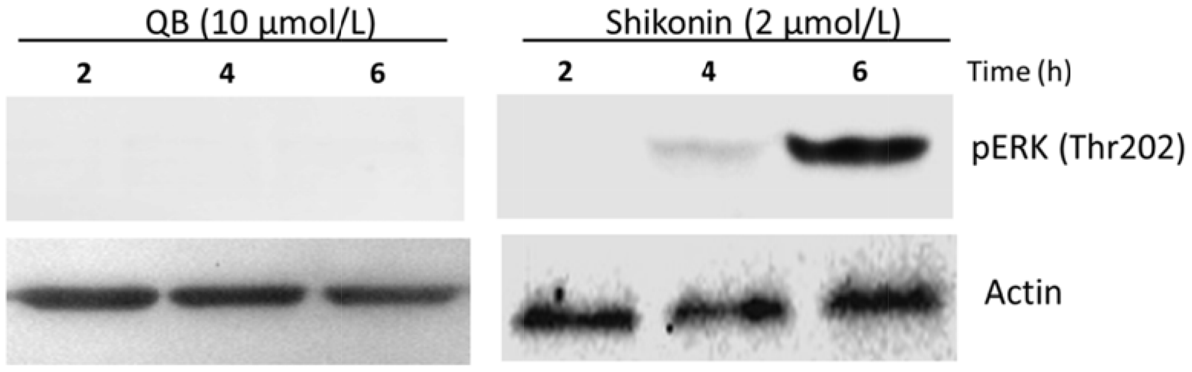

Supplementary Figure 2: Effect of Quambalarine B (QB) and Shikonin on ERK kinase activity in Jurkat cells.
